# Supplementary material for: Heat adaptation of phage T7 under an extended genetic code
Source: Virus Evol. 2021 Dec 1;7(2):veab100. doi: 10.1093/ve/veab100 (PMC8923235; doi:10.1093/ve/veab100)
Supplement: veab100_Supp [file veab100_supp.zip › Sequencing_Analysis.zip!/Sequencing_Analysis/A1/evidence/RA_44.html]

BRESEQ :: Evidence


| Read alignment evidence... | | | | | | | | | | | |
| --- | --- | --- | --- | --- | --- | --- | --- | --- | --- | --- | --- |
|  | seq id | position | | ref | new | freq | score (cons/poly) | reads | annotation | genes | product |
| \* | NC\_001604.1 | 35,515 | 0 | A | C | 22.7% | 2999.6 / inf | 2941 | intergenic (–/–) | *–/–* | –/– |
| Reads supporting (aligned to +/- strand):  **ref** base A (1308/951);  **new** base C (394/271);  **total** (1711/1230) | | | | | | | | | | | |
| Fisher's exact test for biased strand distribution *p*-value = 5.61e-01 | | | | | | | | | | | |
| Kolmogorov-Smirnov test that lower quality scores support variant *p*-value = 1.00e+00 | | | | | | | | | | | |

Only 100 of 2962 total aligned reads displayed.  

`CAGTGGCGCGATGAGACCAAGGGTTTCCGAGACGAAGCCAAGCGGTTCAAGAATACGGCTGGTCAATACGCTACATCTGCTGGGAACTCTGCTTCCGCTGCGCATCAATCTGAGGTAAACGCTGAGAACTCTGCCACAGCATCCGCTAACTCTGCTCATTTGGCAGAACAGCAAGCAGACCGTGCGGAACGTGAGGCAGACAAGCTGGAAAATTACAATGGATTGGCTGGTGCAATTGATAAGGTAGATGGAACCAATGTGTACTGGAAAGGAAATATTCACGCTAACGGGCGCCTTTACATGACCACAAACGGTTTTGACTGTGGCCAGTATCAACAGTTCTTTGGTGGTGTCACTAATCGTTACTCTGTCATGGAGTGGGGAGATGAGAACGGATGGCTGATGTATGTTCAACGTAGAGAGTGGACAACAGCGATAGGCGGTAACATCCAGTTAGTAGTAAACGGACAGATCATCACCCAAGGTGGAGCCATGACCGGTCAGCTAAAATTGCAG‑AATGGGCATGTTCTTCAATTAGAGTCCGCATCCGACAAGGCGCACTATATTCTATCTAAAGATGGTAACAGGAATAACTGGTACA  >  NC_001604.1/35215‑35815`  

                                                                                                                                                                                                                                                                                                            `|`                                                                                                                                                                                                                                                                                                               
  `gtggtgCGATGAGACCAAGGGTTTCAGAGACGAAGCCGAGCGGTTCAAGAATACGGCTGGTCAATACGCTACATCTGCTGGGAACTCTGCTTCCGCTGCGCATCAATCTGAGGTAAACGCTGAGAACTCTGCCACAGCATCCGCTAACTATGCTCATTTGGCAGAACAGCAAGCAGACCGTTCGGAACGTGAGGCAGACAAGCTGGAAAATTACAATGGATTGGCGGGTGCAATTGATATGGTAGATGGAACCAATATGTACTGGAAAGGAAATATTCACGCTACAGGGCGCCTTTACATg                                                                                                                                                                                                                                                                                                             >  1:26872/1‑301 (MQ=255)`  
    `ggCGCGATGAGACCAAGGGTTTCCGAGACGAAGCCAAGCGGTTCAAGAATACGGCTGTTCAATACGCTACATCTGCTGGGAACTCTGCTTCCGCTGCGCATCAATCTGAGGTAAACGCTGAGAACTCTGCCACAGCATCCGCTAACTATGCTCATTTGGCAGAACAGCAAGCAGACCGTGCGGAACGTGATGCAGACAAGCTGGACAATTACAATGGATTGGCTGGTGCAATTGATAAGGTAGATGGAACCAATGTGTACTGGAAAGGAAATATTCACGCTACCGGGCGCCTTTACATGAc                                                                                                                                                                                                                                                                                                           >  1:435519/1‑301 (MQ=255)`  
           `ctagccaaaGGGTTTCCGAAGCTAATCCAAGCTGTTCAAGACTACGTCTGGTCAATACGCTACATCTGCTCGGAGCTATTCTTCCGCTGCGCATCAATCTGAGGTAAACGCCGAAAACTCTGCCACAGCATCCGCTAACTATGCTCATTTGGAAGGACCGCAAGCAGAACGTGCGAAACATGAGGCACACAAGCTGGAAAATTACAATGGATTGGCTGGTGCAATTGATAAGGTAGATGGAACCAATGTGTACTGGAAAGGCAATATTCCCGCTAACGGGCGCCTTTACATGACCACAAAc                                                                                                                                                                                                                                                                                                    <  1:205378/294‑1 (MQ=255)`  
            `gtataagagacagtCCGAGACTAAGCCAAGCGGTTAAAGAAGATGGCTGGTAAATAAGCTACATCTGCTGGGAACTCTGCTTCCGCTGCGCATCAATCTGAGGTAAACGCTGAGAACTCTGCCACAGCATCCGCTAACTATGCTCATTTGGCAGAACAGCAAGCAGACCGTGCGGAACGTGAGGCAGACAAGCTGGAAAATTACAATGGATTGGCTGGTGCAATTGATAAGGTAGATGGAACCAATGTGTACTGGAAAGGAAATATTCACGCTAACGGGCGCCTTTACATGACCACAAACg                                                                                                                                                                                                                                                                                                   <  1:144130/288‑1 (MQ=255)`  
              `gaCCAAGGGTTTCCGAGACGAAGCCAAGCGGTTCAAGAATACGGCTGGTCAATACGCTACATCTGCTGGGAACTCTGCTTCCGCTGCGCATCAATCTGAGGTAAACGCTGAGAACTCTGCCACAGCATCCGCTAACTATGCTCATTTGGCAGAACAGCAAGCAGACCGTGCGGAACGTGAGGCAGACAAGCTGGAAAATTACAATGGATTGGCTGGTGCAATTGATAAGGTAGATGGAACCAATGTGTACTGGAAAGGAAATATTCACGCTAACGGGCGCCTTTACATGACCACAAACGGt                                                                                                                                                                                                                                                                                                 >  1:323220/1‑301 (MQ=255)`  
                    `gggTTTCCGAGACGAAGTCGAGCGGTTCAAGAATACGGCTGGTCAATACGCTACATCTGCTGGGAACTCTGCTTCCGTTGCGCATCAATCTGAGGTAAACGCTGAGAACTCTGCCACAGCATCCGCTAACTATGCTCATTTGGCAGAACAGCAAGCAGACCGTGCGGAACGTGAGGCAGACAAGCTGGAAAATTACAATGGATTGGCTGGTGCAATTGATAAGGTAGATGGAACCAATGTGTACTGGAAAGGAAATATTCACGCTAACGGGCGCCTTTACATGACCACAAACGGTTTTGAc                                                                                                                                                                                                                                                                                           >  1:59688/1‑301 (MQ=255)`  
                    `gggTTTCCGAGACGAAGCCGAGCGGTTCAAGAATACGGCTGGTCAATACGCTACATCTGCTGGGAACTCTGCTTCCGCTGCGCATCAATCTAAGGTAAACGCTGAGAACTCTGCCACAGCATCCGCTAACTCTGCTCATTTGGCAGAACAGCAAGCAGACCGTGCTGAACGTGAGGCAGCCAAGCTGGAAAATTACCATGGATTGGCTGGTGCAATTGCTAAGGTAGATGGAACCAATGTGTACTGGAAAGGAAATATTCACGCTAACGGGCGCCTTTACATGACCACACACGGTTTTGaa                                                                                                                                                                                                                                                                                           >  1:124798/1‑300 (MQ=255)`  
                     `aagtgaccggaCGAAGCCGAGCGGTTAAAGAATACGGCAGGTCAAGACGCTACATCTGCTGGGAACTCTGCTTCCGCTGCGCATCAATCTGAGGTAAACGCTGAGAACTCTGCCACAGCATCCGCTAACTATGCTCATTTGGCAGAACAGCAAGCAGACCGTGCGGAACGTTAGGCAGACAAGCTGGAAAATTACAATGGATTGGCTGGTGCAATTGATAAGGTAGATGGAACCAATGTGTACTGGAAAGGAAATATTCACGCTAACGGGCGCCTTTACATGACCACAAACGGTTTTGACt                                                                                                                                                                                                                                                                                          <  1:331449/292‑1 (MQ=255)`  
                       `tttCCGAGACGAAGCCGAGCGGTTCAAGAATACGGCTGGTCAATACGCTACATCTGCTGGGAACTCTGCTTCCGCTGCGCATCAATCTGAGGTAAACGCTGAGAACTCTGCCACAGCATCTGCTAACTATGCTCATTTGGCAGAACAGCAAGCAGACCGTGCGGAACGTGAGGCAGACAAGCTGGAAAATTACAATGGATTGGCTGGTGCAATTGATAAGGTAGATGGAACCAATGTGTACTGGAAAGGAAATATTCACGCTAACGGGCGCCTTTACATTACCACAAACGGTTGTGACtgt                                                                                                                                                                                                                                                                                        >  1:499157/1‑301 (MQ=255)`  
                           `cgcgaCGAAGCCAATCGTTTCAATAAGACGTCTGCTCAATTCGCTACATCTGCTTGTACCTTTGCTGCCGCTGCGCATCAATCTGAGGTTAACGCTCAGACATCTGCCACAGCACCCGCGAACTATGCTCATTTGGCAGAACAGCAATCTGACTGTGCGGAACGTGAGGCAGACAATCTGGAAAATTACAATGGAGTGGCTGGTGCAATTGATCAGGTAGATGGAACCGATGTGTACTGGAAAGGAAATATTCACGCTAACGGGCGCCTTTACATGACCACAAACGGTTTTGACTGTGGcc                                                                                                                                                                                                                                                                                    <  1:366093/298‑1 (MQ=255)`  
                             `agaCGAAGCCGAGCGGTTCAAGAATACGGCTGGTCAATACGCTACATCTGCTGGGAACTCTGCTTCCGCTGCGCATCAATCTGAGGTAAACGTTGAGAACTCTGCCACAGCATCCGCTAACTATGCTCATTTGGCAGAACAGCAAGCAGACCGTGCGGAACGTGAGGCAGACAAGCTGGAAAATTACAATGGATTGGCTGGTGCAATTGATAAGGTAGATGGAACCAATGTGTACTGGAAAGGAAATATTCACGCTAACGGGCGCCTTTACATGACCACAAACGGTTTTGACTGTGGCCAg                                                                                                                                                                                                                                                                                  >  1:294387/1‑301 (MQ=255)`  
                                    `gCCAAGCGGTTCAAGAATACGGCTGGTCAATTCGCTACATCTGCTTGGAACTCTGCTTCCGCTGCGCATCAATCTGAGGTAAACGCTGAGAACTCTGCCACAGCATCCGCTAACTATGCTCATTTGGCAGAACAGCAAGCAGACAGTGCGGAACGTGAGGCAGACAAGCTGGAAAATTACAATGGATTGGCTGGTGCAATTGATAAGGTAGATGGAACCAATGTGTACTGGAAAGGAAATATTCACGCTAACGGGCGCCTTTACCTGACCACAAACGGTTTTGACTGTGGCCAGTATCAAc                                                                                                                                                                                                                                                                           <  1:64861/301‑1 (MQ=255)`  
                                            `gTTCAAGAATACGGCTGGTCAATACGCTACATCTGCTTGGAACTCTGCTTCCGCTGCGCATCAATCTGAGGTAAACGCTGAGAACTCTGCCACAGCATCCGCTAACTATGCTCATTTGTCAGAACAGAAAGCAGACCGTGCGGAACGTGAGGCAGACAAGCTGGAAAATTACAATGGATTGGCTGGTGCAATTGATAAGGTAGCTGGAACCAATGTGTACTGGAAAGGAAATATTCACGCTAACGGGCGCCTTTACATGACCACCAACGGTTTTGCCTTTTGCCAGTATCAACAGTTCttt                                                                                                                                                                                                                                                                   >  1:67560/1‑301 (MQ=255)`  
                                            `gTTCAAGAATACGGCTGGTCAATACGCTACATCTGCTGGGAACTCTGCTTCCGCTGCGCATCAATCTGAGGTAAACGCTGAGAACTCTGCCACAGCATCCGCTAACTCTGCTCATTTGGCAGAACAGCAAGCAGACCGTGCGGAACGTGAGGCAGACAAGCTGGAAAATTACAATGGATTGGCTGGTGCAATTGATAAGGTAGATGGAACCAATGTGTACTGGAAAGGAAATATTCACGCTAACGGGCGCCTTTACATGACCACAAACGGTTTTGACtgctgtctcttatacacatctccc                                                                                                                                                                                                                                                                   >  1:412368/1‑279 (MQ=255)`  
                                            `gTTCAAGAATACGGCTGGTCAATACGCTACATCTGCTGGGAACTCTGCTTCCGCTGCGCATCAATCTGAGGTAAACGCTGAGAACTCTGCCACAGCATCCGCTAACTATGCTCATTTGGCAGAACAGCAAGCAGACCGTGCGGAACGTGAGGCAGACAAGCTGGAAAATTACAATGGATTGGCTGGTGCAATTGATAAGGTAGATGGAACCAATGTGTACTGGAAAGGAAATATTCACGCTAACGGGCGCCTTTCCATGACCACAAACTGTTTTTACTTTGGCCAGTATCAACAGTTCttt                                                                                                                                                                                                                                                                   >  1:327446/1‑301 (MQ=255)`  
                                              `tCAAGAATACTGCTTGTCAATACGCTACATCTGCTGGGAACTCTGCTTCCGCTGCGCATCAATCTGAGGTAAACGCTTAGAACTCTGTCACAGCATCCGCTAACTATGCTCATTTGGCAGAACAGCAAGCAGACCGTGCGGAACGTGAGGCAGACAAGCTGGAAAATTACAATGGATTGGCTGGTGCAATTGATAAGGTAGATGGAACCAATGTGTACTGGAAAGGAAATATTAACGCTAACGGGCGCCTTTACATGACCACAAACGGTTTTGACTGTGGCCAGTATCAACAGTTCTTtgg                                                                                                                                                                                                                                                                 <  1:15663/301‑1 (MQ=255)`  
                                                  `gAATATGGCTGGTCAATACGCTACATCTGCTGGGAACTCTGCTTCCGCTGCGCATCAATCTGAGGTAAACGCTGAGAACTCTGCCACAGCATCCGCTAACTATGCTCATTTGGCAGAACAGCAAGCAGACCGTGCGGAACGTGAGGCAGACAAGCTGGAAAATTACAATGGATTGGCTGGTGCAATTGATAAGGTAGATGGAACCAATGTGTACTGGAAAGGAAATATTCACGCTAACGGGCGCCTTTACATGACCACAAACGGTTTTTACTGTGGCCAGTATCAACAGTGCTTtggtggg                                                                                                                                                                                                                                                             >  1:263307/1‑300 (MQ=255)`  
                                                       `cGGCTGGTCAATACGCTACATCTGCTGGGAACTCTGCTTCCGCTGCGCATCAATCTGAGGTAAACGCTGAGAACTCTGCCACAGCATCCGCTAACTATGCTCATTTGGCAGAACAGCAAGCAGACCGTGCGGAACGTGAGGCAGACAAGCTGGAAAATTACAATGGATTGGCTGGTGCAATTGATAAGGTAGATGGAACCGATGTGTACTGGAAAGGAAATATTCACGCTAACGGGCGCCTTTACATGACCACAAACGGTTTTGACTGTGGCCAGTATCAACAGTTCTTTGGTGGTGTAAc                                                                                                                                                                                                                                                        >  1:53643/1‑301 (MQ=255)`  
                                                            `gggcAATACTCTACATCTGCTGGGAACTCATCTTCCGCTGCGCATCAATCTGAGGTAAACGCTGAGAACTCCGCCACAGCATCCGCTAACTATGCTTATTTGGCAGAACAGCAAGCAGATCGTGCGGAACGTGAGGCAGACAATCTGGAAAATTACAATGGAGTGGCTGGTGCAATTGATAAGGTAGATGGAACCAATGTGTACTAGAAAGGAAATATTCACGCTAACGGGCGCCTTTACATGACCACAAACGGTTTTGACTGTGGCCAGTATCAACAGTTCTTTGGTGGTGTCACTAATc                                                                                                                                                                                                                                                   <  1:78462/298‑1 (MQ=255)`  
                                                                 `aTACGCTACATCTGCTGGGAACTCTGCTTCCGCTGCGCATCAATCTGAGGTAAACGCTGAGAACTCTGCCACAGCATCCGCTAACTATGCTCATTTGGCAGAACAGCAAGCAGACCGTGCGGAACGTGAGGCAGACAAGCTGGAAAATTACAATGGATTGGCTGGTGCAATTGATAAGGTAGATGGAACCAATGTGTACTGGAAAGGAAATATTCACGCTAACGGGCGCCTTTACATGACCACAAACGGTTTTGACTGTGGCCAGTATAAACAGTTCTTTGGTGGTGTCACTAATCGTTAc                                                                                                                                                                                                                                              >  1:252925/1‑301 (MQ=255)`  
                                                                 `aTACGCTACATCTGCTGGGAACTCTGCTTCCGCTGCGCATCAATCTGAGGTAAACGCTGAGAACTCTGCCACAGCATCCGCTAACTATGCTCATTTGGCAGAACAGCAAGCAGACCGTGCGGAACGTGAGGCAGACAAGCTGGAAAATTACAATGGATTGGCTGGTGCAATTGATAAGGTAGATGGAACCAATGTGTACTGAAAAGGAAATATTCACGCTAACGGGCGCCTTTACATGACCACAAACGGTTTTGACTGTGGCCAGTATCAACAGTTCTTTGGTGGTGTCACTAATCGTTAc                                                                                                                                                                                                                                              <  1:220200/301‑1 (MQ=255)`  
                                                                   `aagCTACATCTGGTGGGAACTCTGCTTCCGCTGCGCATCAATCTGAGGTAAACGCTGAGAACTCTGCCACAGCATCCGCTAACTATGCTCATTTGGCAGAACAGCAAGCAGACCGTGCGGAACGTGAGGCATACAAGCTGCAAAATTACAATGGATTGGCTGGTGCAATTGATAAGGTAGATGGAACCAATGTGTACTGGAAAGGAAATATTCACGCTAACGGGCGCCTTTACATGACCACAAACGGTTTTGACTGTGGCCAGTATCAACAGTTCTTTGGTGGTGTCACTAATCGTTActc                                                                                                                                                                                                                                            <  1:431302/299‑1 (MQ=255)`  
                                                                   `aCGCTACATCTGCTGGGAACTCTGCTTCCGCTGCGCATCAATCTGAGGTAAACGCTGAGAACTATGCCACAGCATCCGCTAACTATGCTCATTTGGCAGAACAGCAAGCAGACCGTGCGGAACGTGAGGCAGACAAGCTGGAAAATTACAATGGATTGGCTGGTGCAATTGATAAGGTAGATGGAACCAATGTGTACTGGAAAGGAAATATTCACGCTAACGGGCGCCTTTACATGACCACAAACGGTTTTGACTGTGGCCAGTATCAACAGTTCTTTGGTGGTGTCACTAATCGTTActc                                                                                                                                                                                                                                            >  1:167905/1‑301 (MQ=255)`  
                                                                    `cGCTACATCTGCTGGGAACTCTGCTTCCGCTGCGCATCCATCTGAGGTCAACGATGAGAACTCTGCCACAGCATCCGCTAACTATGCTCATTTGGCAGAACAGCAAGCAGACCGTGCGGAACGTGAGGCAGACAAGCTGGAAAATTACAATGGATTGGCTGGTGCAATTGATAAGGTAGATGGAACCAATGTGTACTGGAAAGGAAATATTCACGCTAACGGGCGCCTTTACATGACCACAAACGGTTTTGACTGTGGCCAGTATCAACAGGTCTTTGGTGGTGTCACTAATCGTTActct                                                                                                                                                                                                                                           >  1:491332/1‑301 (MQ=255)`  
                                                                    `cGCTACATCTGCTGGGAACTCTGCTTCCGCTGCGCATCAATCTGAGGTAAACGCTGAGAACTCTGCCACAGCATCCGCTAACTATGCTCATTTGGCAGAACAGCAAGCAGACCGTGCGGAACGTGCGGCAGACAAGCTGGAAAATTACAATGGATTGGCTGGTGCAATTGATAAGGTAGATGGAACCAATGTGTACTGGAAAGGAAATATTCACGCTAACGGGCGCCTTTACATGACCACAAACGGTTTTGACTGTGGCCAGTATCAACAGTTCTTTGGTGGTGTCACTAATCGTTActct                                                                                                                                                                                                                                           >  1:427227/1‑301 (MQ=255)`  
                                                                           `tCTGCTGGGAACTCTGCTTCCGCTGCGCATCAATCTGAGGTAAACGCTGAGAACTCTGCCACAGCATCCGCTAACTATGCTCATTTGGCAGAACAGCAAGCAGACCGTGCGGAACGTGAGGCAGACAAGCTGGAAAATTACAATGGATTGGCTGGTGCAATTGATAAGGTAGATGGAACCAATGTGTACTGGAAAGGAAATATTCACGCTAACGGGCGCCTTTACATGACCACAAACGGTTTTGACTGTGGCCAGTATCAACAGTTCTTTGGTGGTGTCACTAATCGTTACTCTGTCATgg                                                                                                                                                                                                                                    >  1:375570/1‑301 (MQ=255)`  
                                                                            `ctgctgGGAACTCTGCTTCCGCTGCGCATCAATCTGAGGTAAACGCTGAGAACTCTGCCACAGCATCCGCTAACTATGCTCATTTGGCAGAACAGCAAGCAGACCGTGCGGAACGTGAGGCAGACAAGCTGGAAAATTACAATGGATTGGCTGGTGCAATTGATAAGGTAGATGGAACCAATGTGTACTGGAAAGGAAATATTCACGCTAACGGGCGCCTTTACCTGACCACAAATGGTTTTGACTGTGGCCAGTATCAACAGTTCTTTGGTTGTGTCCCTAATCgctgtctcttatacac                                                                                                                                                                                                                                   >  1:224624/1‑286 (MQ=255)`  
                                                                             `tgctgGGAACTCTGCTTCCGCTGCGCATCAATCTGAGGTAAACGCTGAGAATTCTGCCACAGCATCCGCTAACTATGCTCATTTTGCAGAACAGCAAGCAGACCGTGCGGAACGTGAGGCAGACAAGCTGGAAAATTACAATGGATTGGCTGGTGCAATTGATAAGGTAGATGGAACCAATGTGTACTGGAAAGGAAATATTCACGCTAACGGGCGCCTTTACCTGACCACAAACGGTTTTGACTGTGGCCAGTATCAACAGTTCTTTGGTGGTGTCACTAATCGTTACTCTGTCATGGAg                                                                                                                                                                                                                                  <  1:115964/301‑1 (MQ=255)`  
                                                                             `tgctgGGAACTCTGCGTCCGCGGCGCATCAATGTGAGGTAAATGCTGAGAACTCTGCTACAGCATCAGCTAACTCTGCTCATTGGGCAGAACAGCGAGCAGACCGTGCGGAACGTGAGGCAGACAAGCTGGAAAATTACCATGGATTGGCTGGTGCAATTGATAAGGTAGATGGAACCGATGTGTACTGGAAAGGAAATATTCACGCTAACGGGCGCCTTTACATGACCACAAACGGTTTTGACTGTGGCCAGTATCAACAGTTCTTTGGTGGTGTCACTAATCGTTACTCTGTCATGGAg                                                                                                                                                                                                                                  <  1:362584/301‑1 (MQ=255)`  
                                                                                   `gAACTCTGCTTCCGCTGCGCATCAATCTGAGGTAAACGCTGAGAACTCTGCCACAGCATCCGCTACCTATGCTCATTTGGCAGAACAGCAAGCAGACCGTGCGGAACGTTAGGCAGACAAGCTGGAAAATTACAATGGATTGGCTGGTGCAATTGATAAGGTAGATGGAACCAATGTGTACTGGAAAGGAAATATTCACGCTAACGGGCGCCTTTACATGACCACAAACGGTTTTGACTGTGGCCAGTATCAACAGTTCTTTGGTGGTGTCACTAATCGTTACTCTGTCATGGAGTggggg                                                                                                                                                                                                                            >  1:485034/1‑300 (MQ=255)`  
                                                                                   `gAACTCTGCTTCCGCTGCGCATCAATCTGAGGTAAACGCTGAGAACTCTGCCACAGCATCCGCTAACTATGCTCATTTGGCAGAACAGCAAGCAGACCGTGCGGAACGTGAGGCAGACAAGCTGGAAAATTACAATGGATTGGCTGGTGCAATTGATAAGGTAGATGGAACCAATGTGTACTGGAAAGGAAATATTCACGCTAACGGGCGCCTTTACATGACCACAAACGGTTTTGACTGTGGCCAGTATCAACAGTTCTTTGGTGGTGTCACTAATCGTTACTCTGTCATGGAGTGGTga                                                                                                                                                                                                                            >  1:120582/1‑301 (MQ=255)`  
                                                                                   `gAACTCTGCTTCCGCTGCGAATCAATCTGAGGTAAACGCTGAGAACTCTGCCACAGCATCCACTAACTCTGCTCATTTGGCAGAACAGCAAGCAGACCGTGCGGAACGTGAGGCAGACAAGCTGGAAAATTACAATGGATTGGCTGGTGCAATTGATAAGGTAGATGGAACCGATGTGTACTGGAAAGGAAATATTCACGCTAACGGGCGCCTTTACATGACCACAAACGGTTTTGACTGTGGCCAGTATCAACAGTTCTTTGGTGGTGTCACTAATCGTTACTCTGTCATGGAGTGGGga                                                                                                                                                                                                                            <  1:417816/301‑1 (MQ=255)`  
                                                                                    `aaatctgcctgcgcgtcacatcagtagttggTAAAGGCTAAGAACTATGCCAACGCAAACGCTAACTACGCTCAGATGGCAGAACAGCAAGCAGACCGTGCGGACTGTGAGTCAGACAAGCTGGAAAATTACAATGGATTGGCTGGTGCAATTGATAAGGTAGAAAGAACCAATGTGTACTGGAAAGGAAATATTCACACTAACGGGAGCCTTTACATGACCACAAACGGTTTTGACTGTGGACAGTATCAAAAGTTCTTTGGTGGAGTCACTAATCGTTACTCTGTCATGGAGTGGGgag                                                                                                                                                                                                                           <  1:110567/272‑1 (MQ=255)`  
                                                                                    `aaCTCTGCTTCCGCTGCGCATCAATCTGAGGTAAACGCTGAGAACTCTGCCACAGCATCCGCTAACTATGCTCATTTGGCAGAACAGCAAGCAGACCGTGCGGAACGTGAGGCAGACAAGCTGGAAAATTACAATGGATTGGCTGGTGCAATTGTTAAGGTAGATGGAACCAATGTGTACTGGAAAGGAAATATTCACGCTAACGGGCGCCTTTACATGACCACAAACGGTTTTGACTGTGGCCAGTATCAACAGTTCTTTGGTGGTGTCACTAATCGTTACTCTGTCATGGAGTGGGgag                                                                                                                                                                                                                           <  1:216672/301‑1 (MQ=255)`  
                                                                                     `aCTCTGCTTCCGCTGCGCATCAATCTGAGGTAAACGCTGAGAACTCTGCCACAGCATCCGCTAACTATGCTCATTTGGCAGAACAGCAAGCAGAACGTGCGGAACGTGAGGCAGACAAGCTGGAAAATTACAATGGATTGGCTGGTGCAATTGATAAGGTAGATGGAACCAATGTGTACTGGAAAGGAAATATTCACGCTAACGGGCGCCTTTACATGACCACAAACGGTTTTGACTGTGGCCAGTATCAACAGTTCTTTGGTGGTGTCACTAATCGTTACTCTGTCATGGAGTGGGgaga                                                                                                                                                                                                                          <  1:401146/301‑1 (MQ=255)`  
                                                                                                   `gcgcATCACTCTGAGGTAAACGCTGAGAACTATGCCACAGCATCCGCTAACTCTGCTCATTTGGCAAAACAGCAAGCAGACCGTGCGGAACGTGAGGCAGACAAGCTGGAAAATTACAATGGATTGGCTGGTGCAATTGATAAGGTAGATGGAACCAATGTGTACTGGAAAGGAAATATTCACGCTAACGGGCGCCTTTACATGACCACAAACGGTTTTGACTGTGGCCAGTATCAACAGTTCTTTGGTGGTGTCACTAATCGTTACTCTGTCATGGAGTGGGGAGATGAGAACGGATggg                                                                                                                                                                                                            >  1:471862/1‑300 (MQ=255)`  
                                                                                                   `gcgcATCAATCTGAGGTAAACGCTGAGAACTCTGCCACAGCATCCGCTAACTATGCTCATTTGGCAGAACAGCAAGCAGACCGTGCGGAACGTGAGGCAGACAAGCTGGAAAATTACAATGGATTGGCTGGTGCAATTGATCAGGTAGATGGAACCAATGTGTACTGGAAAGGAAATATTCACGATAACGGGCGCTTTTACATGACCACAAACGGTTTTGACTGTGGCCAGTATCAACAGTTCTTTGGTGGTGTCACTAATCGTTACTCTGTCATGGAGTGGGGAGATTAGAACGGATGGc                                                                                                                                                                                                            >  1:277377/1‑301 (MQ=255)`  
                                                                                                   `gcgcATCAATCTGAGGTAAACGCTGAGAACTCTGCCACAGCATCCGCTAACTATGCTCATTTGGCAGAACAGCAAGCAGACCGTGCGGAACGTGAGGCAGACAAGCTGGAAAATTACAATGGATTGGCTGGTGCAATTGATAAGGTAGATGGAACCAATATGTACTGGAAAGGAAATATTCACGCTAACGGGCGCCTTTACCTGACCACAAACGGTTTTGACTGTGGCCAGTATCAACAGTTCTTTGGTGGTGTCACTAATCGTTACTCTGTCATGGAGTGGGGAGATGAGaaactgtctc                                                                                                                                                                                                            >  1:202274/1‑293 (MQ=255)`  
                                                                                                           `aTCTTAGGTAAACGCTGAGAACTCTGCCACAGCATCCGCTAACTATGCTCATTTGGCAGAACAGCAAGCAGACCGTGCGGAACGTGAGGCATACAAGCTGGAAAATTACAATGGATTGGCTGGTGCAATTGATACGGTAGATGGAACCAATGTGTACTGGAAAGGAAATATTCACGCTAACGGGCGCCTTTACATGACCACAAACGGTTTTGACTGTGGCCAGTATCAACAGTTCTTTGGTGGTTTCCCTAATCGTTACTCTGTCATGGCGTGGGGAGATGAGAACGGATGGCTGatgtat                                                                                                                                                                                                    >  1:152134/1‑301 (MQ=255)`  
                                                                                                           `aTCTGAGGTAAACGCTGAGAACTCTGCCACAGCATCCGCTAACTATGCTCATTTGGCAGAACAGCAAGCAGACCGTGCGGAACGTGAGGCAGACAAGCTGGAAAATGAAAAGGGATTGGCTGGTGCAATTGATAAGGTAGATGGAACCAATGTGTACTGGAAAGGAAATATTCAAGCTAACGGGCGCCTTTACCTGACCACAAACGGTTGTGACTGTGGCAAGTATCAACAGTTCTTTGGTGGTGTCACTAATGGTTACTCTGTCATGGAGTGGGTGGATGAGAACGGGTGGGTGatgtag                                                                                                                                                                                                    >  1:100/1‑300 (MQ=255)`  
                                                                                                               `tgtataagagacaggaACTCTGCCACAGCATCCGCTAACTATGCTCATTTGGCAGAACAGCAAGCAGACCGTGCGGAACGTGAGGCAGACAAGCTGGAAAATTACAATGGATTGGCTGGTGCAATTGATAAGGTAGATGGAACCAATGTGTACTGGAAAGGAAATATTCACGCTAACGGGCGCCTTTACATGACCACAAACGGTTTTGACTGTGGCCAGTATCAACAGTTCTTTGGTAGTGTCACTAATCGTTACTATGTCATGGAGTGGGGAGATGAGAACGGATGGCTGATGTATGTTc                                                                                                                                                                                                <  1:147905/287‑1 (MQ=255)`  
                                                                                                               `agggTAAACGCTGAGAACTCTGCCACAGCATCCGCTAACTCTGCTCATTTGGCAGAACAGCAAGCAGACAGTGCGGAACGTGAGGCAGACAAGCTGGAAAATTACAATGGATTGGCTGGTGCAATTGATAAGGTAGATGGAACCGATGTGTACTGGAAAGGAAATATTCACGCTAACGGGCGCCTTTACATGACCACAAACGGTTTTGACTGTGGCCAGTATCAACAGTGCTTTTGTGGTGTCACTAATCGTTACTCTGTCATGGAGTGGGGAGATGAGAACGGATGGCTGATGTATGTTc                                                                                                                                                                                                <  1:408260/299‑1 (MQ=255)`  
                                                                                                                  `gtgggctcgtagatgtgtatcagcgacatcGCTAACTATGCTCATTTGTCAGAACAGCAAGCAGACCGTGCGGAACGTGATGCAGACAAGCTTGAAAATTACAATGGATTGGCTGGTGCAATTGATAAGGTAGATGGAACCAATGTTTACTGGAAAGGAAATATTCACGCTAACGGGCGCCTTTACCTGACCACAAACGGTTTTGACTGTGGCCAGTATCAACAGTTCTTTGGTGGTGTCACTAATCGTTACTCTGTCATGGAGTGGGGAGATGAGAACGGATGGCTGATGTATGTTCAAc                                                                                                                                                                                             <  1:386989/272‑1 (MQ=255)`  
                                                                                                                    `aaaaGCTGAGAACTCTACCATCGCATCCGCTAAATAAGCTCAATTGGCAGAACAGCAAGCAGACCGGGCGTAACGTGAGGCAGACAAGCTGGAAAATTACAATGGATTGGCTTGTGCAATTGAGAAGGTAGATGGAACCAATGTGTACTGGAAAGGAAATATTCACGCTAACGGGCGCCTTTACATGACCACAAACGGTTTTGACTGTGGCCAGTATCAACAGTTCTTTGGTGGTGTCACTAATCGTTACGATGTCATGGAGTGGGGAGATGAGAACGGATGGCTGATGTATGTTCAACGt                                                                                                                                                                                           <  1:258246/301‑1 (MQ=255)`  
                                                                                                                       `cGCTGAGAACTCTGCCACAGCATCCGCTAACTCTGCTCATTTGGCAGAACAGCAAGCAGACCGTGCGGAACGTGAGGCAGACAAGCTGGAAAATTACAATGGATTGGCTGGTGCAATTGATAAGGTAGATGGAACCGATGTGTACTGGAAAGGAAATATTCACGCTAACGTGCGCCTTTACATGACCACAAACGGTTTTTACTGTGGCCAGTATCAACAGTTCTTTGGTGGTGTCACTAATCGTTACTCTGTCATGGAGTGGGGAGATGAGAACGGATGGCTGATGTATGTTCAACGTaga                                                                                                                                                                                        >  1:396608/1‑301 (MQ=255)`  
                                                                                                                        `tataagagacaggCCACAGCATCCGCTAACTCTGCTCATTTGGCAGAACAGCAAGCAGACCGTGCGGAACGTGAGGCAGACAAGCTGGAAAATTACAATGGATTGGCTGGTGCAATTGATAAGGTAGATGGAACCAATGTGTACTGGAAAGGAGATATTCACGCTAACGGGCGCCTTTACATGACCACAAACGGTTTTGACTGTGGCCAGTATCAACAGTTCTTTGGTGGTGTCACTAATCGTTACTCTGTCATGGAGTGGGGAGATGAGAACGGATGGCTGATGTATGTTCAACGTagag                                                                                                                                                                                       <  1:443443/289‑1 (MQ=255)`  
                                                                                                                             `gaACTCTGCCACAGCATCCGCTAACTATGCTCATTTGGCAGAACAGCAAGCAGACCGTGCGGAACGTGAGGCAGACAAGCTGGAAAATTACAATGGATTGGCTGGTGCAATTGATAAGGTAGATGGAACCAATGTGTACTGGAAAGGAAATATTCACGCTAACGGGCGCCTTTACATGACCACAAACGGTTTTGACTGTGGCCAGTATCAACAGTTCTTTGGTGGTGTCACTAATCGTTACTCTGTCATGGAGTGGGGAGTTGAGAACGGATGGCTGATGTATGTTCAACGTAGAGAGTgg                                                                                                                                                                                  >  1:173269/1‑301 (MQ=255)`  
                                                                                                                               `aCTCTGCCACAGCATCCGCTAACTATGCTCATTTGGCAGAACAGCAAGCAGACCGTGCGGAACGTGAGGCAGACAAGCTGGAAAATTACAATGGATTGGCTGGTGCAATTGATAAGGTAGATGGAACCAATGTGTACTGGAAAGGAAATATTCACGCTAACGGGCGCCTTTACATGACCACAAACGGTTTTGACTGTGGCCAGTATCAACAGTTCTTTGGTGGTGTCACTAATCGTTACTCTGTCATGGAGTGGGGAGATGAGAACGGATGGCTGATGTATGTTCAACGTAGAGAGTGGac                                                                                                                                                                                <  1:463251/301‑1 (MQ=255)`  
                                                                                                                                    `gCCACAGCATCCGCTAACTATGCTCATTTTGCAGAACAGCAAGCAGACCGTGCGGAACGTGAGGCAGACAAGCTGGAAAATTACAATGGATTGGCTGGTGCAATTGATAAGGTAGATGGAACCAATGTGTACTGGAAAGGAAATATTCACGCTAACGGGCGCCTTTACATGACCACAAACGGTTTTGACTGTGGCCAGTATCAACAGTTCTTTGGTGGTGTCAATAATCGTTACTCTGTCATGGAGTGGGGAGATGAGAACGGATGGCTGATGTATGTTCAACGTAGAGAGTGGACAACAg                                                                                                                                                                           <  1:160639/301‑1 (MQ=255)`  
                                                                                                                                            `aTCCGCTAACTATGCTCATTTGGCAGAACAGCAAGCAGACCGTGCGGAACGTGAGGTAGACAAGCTGGAAAATTACAATGGATTGGCTGGTGCAATTGATAAGGTAGATGGAACCAATGTGTACTGGAAAGGAAATATTCACGCTAACGGGCGCCTTTACATGACCACAAACGGTTTTGACTGTGGCCAGTATCAACAGTTCTTTGGTTGTGTCACTAATCGTTACTCTGTCATGGAGTGGGGAGATGAGAACGGATGGCTGATGTATGTTCAACGTAGAGAGTGGACAACAGCGATAGGc                                                                                                                                                                   <  1:85246/301‑1 (MQ=255)`  
                                                                                                                                             `tCCGCTAACTATGCTCATTTGGCAGAACAGCAAGCAGACCGTGCGGAACGTGAGGCAGACAAGCTGGAAAATTACAATGGATTGGCTGGTGCAATTGATAAGGTAGATGGAACCAATGTGTACTGGAAAGGAAATATTCACGCTAACGGGCGCCTTTACATGACCACAAACGGTTTTGACTGTGGCCAGTATCAACAGTTCTTTGGTGGTGTCACTAATCGTTACTCTGTCATGGAGTGAGGAGATGAGAACGGATGGCTGATGTATGTTCAACGTagagatctgtctcttatacacatct                                                                                                                                                                  >  1:505899/1‑281 (MQ=255)`  
                                                                                                                                                  `tAACTATGCTCATTTGGTAGAACAGGAAGCAGACCGGTCGGAACGTGAGGCAGACAAGCTGGAAAATTACAATGGATTGGCTGGTGCAATTGATAAGGTAGATGGAACCAATGTGTACTGGAAAGGAAATATTCACGATAACGGGCGCCTTTACATGACCACAAACGGTTTTGACTGTGGCCAGTATCAACAGTTCTTTGGTGGTGTCACTAATCGTTACTCTGTCATGGAGTGGGGAGATGAGAACGGATGGCTGATGTATGTTCAACGTAGAGAGTGGACAACAGCGATAGGAGGTAAc                                                                                                                                                             <  1:186479/301‑1 (MQ=255)`  
                                                                                                                                                     `ctatGCTCCTTTGGCAGAACAGCAAGCAGACCGTGCGGAACGTGAGGCAGACAAGCTGGAAAATTACAATGGATTGGCTGGTACAATTGATAAGGTAGATGGAACCAATGTGTACTGGAAAGGAAATATTCACGCTAACGGGCGCCTTTACATGACCACAAACGGTTTTGACTGTGGCCAGTATCAACAGTTCTTTGGTGGTGTCACTAATCGTTACTCTGTCATGGAGTGGGGAGATGAGAACGGATGGCTGATGTATGTTCAACGTAGAGAGTGGACAACAGCGATAGGCGGTAACATc                                                                                                                                                          >  1:391977/4‑301 (MQ=255)`  
                                                                                                                                                          `cTCAGTTGGCAGAACAGCAAGCAGACCGTGCGGAACGTGAGGCAGACAAGCTGGAAAATTACAATGGATTGGCTGGTGCAATTGATAAGGTAGATGGAACCAATGTGTACTGGAAAGGAAATATTCACGCTAACGGGCGCCTTTACATGACCACAAACGGTTTTGACTGTGGCCAGTATCAACAGTTCTTTGGTGGTGTCACTAATCGTTACTCTGTCATGGAGTGGGGAGATGAGAACGGATGGCTGATGTATGTTCAACGTAGAGAGTGGACAACAGCGATAGGCGGTAACATCCAGtt                                                                                                                                                     <  1:282544/301‑1 (MQ=255)`  
                                                                                                                                                            `cATTTGGCAGAACAGCAAGCAGACCGTGCGGAACGTGAGGCAGACAAGCTGGAAAATTACAATGGATTGGCTGGTGCAATTGATAAGGTAGATGGAACCAATGTGTACTGGAAAGGAAATATTCTCGCTAACGGGCGCCTTTACATGACCACACACGGTTTTGACTGTGGCCAGTATCAACAGTTCTTTGGTGGTGTCACTAATCGTTACTCTGTCATGGAGTGGGGAGATGAGAACGGATGGCTGATGTATGTTCAACGTAGAGAGTGGACACCAGCGATAGGCGGTAACATCCAGttct                                                                                                                                                   >  1:371236/1‑299 (MQ=255)`  
                                                                                                                                                            `cATTTGGCAGAACAGCAAGCAGACCGTGCGGAACGTGAGGCAGACAAGCTGGAAAATTACAATGGATTGGCTGGTGCAATTGATAAGGTAGAAGGAACCAATGTGTACTGGAAAGGAAATATTCACGCTAACGGGCGACTTTACATGACCACAAACGGTTTTGACTGTGGCACGTATCAACAGTTCTTTGGTGGTGTCACTAATCGTTACCCTGTCATGGAGTGGGGAGATGAGAACGGATGGCTGATGTATGTTCAACGTAGAGAGTGGACAACAGCGATAGGCGGTAACATCCAGTtag                                                                                                                                                   >  1:229481/1‑301 (MQ=255)`  
                                                                                                                                                               `gggctcggagatgtgtataagagacaggAACGTGAGGCAGACAAGCTGGAAAATTACAATGGATTGGCTGGTGCAATTGATAAGGTAGATGGAACCAATGTGTACTGGAAAGGAAATATTCACGCTAACGGGCGCCTTTACATGACCACAAACGGTTTTGACTGTGGCCAGTATCAACAGTTCTTTGGTGGTGTCACTAATCGTTACTCTGTCATGGAGTGGGGAGATGAGAACGGATGGCTGATGTATGTTCAACGTAGAGAGTGGACAACAGCGATAGGCGGTAACATCCAGTtagtag                                                                                                                                                <  1:19963/275‑1 (MQ=255)`  
                                                                                                                                                                `tGGCAGAACAGCAAGCAGACCGTGCGGAACGTGAGGCAGACAAGCTGGAAAATTACAATGGATTGGCTGGTGCAATTGATAAGGTAGATGGAACCAATGTGTACTGGAAAGGAAATATTCACGCTAACGGGCGCCTTTACATGACCACAAACGGTTTTGACTGTGGCCAGTATCAACAGTTCTTTGGTGGTGTCACTAATCGTTACTCTGTCATGGAGTGGGGAGATGAGAACGGATGGCTGATGTATGTTCAACGTAGAGAGTGGACAACAGCGATAGGCGGTAACATCCAgctgtctat                                                                                                                                               >  1:314008/1‑293 (MQ=255)`  
                                                                                                                                                                  `gCAGAACAGCAAGCAGACCGTGCGGAACGTGAGGCAGACAAGCTGGAAAATTACAATGGATTGGCTGGTGCAATTGATAAGGTAGATGGAACCAATGTGTACTGGAAAGGAAATATTCACGCTAACGGGCGCCTTTACATGACCACAAACGGTTTTGACTGTGGCCAGTATCAACAGTTCTTTGGTGGTGTCACTAATCGTTACTCTGTCATGGAGTGGGGAGATGAGAACGGATGGCTGATGTATGTTCAACGTAGAGAGTGGACAACAGCGATAGGCGGTAACATCCAGttctgtctct                                                                                                                                             >  1:447669/1‑293 (MQ=255)`  
                                                                                                                                                                  `gCAGAACAGCAAGCAGACCGTGCGGAACGTGAGGAAGACAAGCTGGAAAATTACAATGGATTGGCTGGTGCAATTGATAAGGTAGATGGAACCAATGTGTACTGGAAAGGAAATATTCACGCTAACGGTCGCCTTTACATGACCACAAACGGTTTTGACTGTGGCCAGTATCAACAGTTCTTTGGTGGTGTCACTAATCGTTACTCTGTCATGGAGTGGGGAGATGAGAACGGATGGCTGATGTATGTTCAACGTAGAGAGTGGACAACAGCGATAGGCGGTAACATCCAGTTAGTAGTaa                                                                                                                                             >  1:81667/1‑301 (MQ=255)`  
                                                                                                                                                                    `aagaCAGCAAGAAGAACGTGCGGAACGTGAGGCAGACAAGCTGGAAAATTACAATGGATTGGGTGGTGCAATTGATAAGGTAGATGGAACCAATGTGTACTGGAAAGGAAATAGTCACGATAACGGGCGCCTTTAAATGACCACAAACGGTTTTGACTGTGGCCAGTATCAACAGTTCTTTGGTGGTGTCACTAATCGTTACTCTGTCATGGAGTGGGGAGATGAGAACGGATGGCTGATGTATGTTCAACGTAGAGAGTGGACAACAGCGATAGGCGGTAACATCCAGTTAGTAGTAAAc                                                                                                                                           <  1:177653/298‑1 (MQ=255)`  
                                                                                                                                                                          `gcaagcaGACCGTGCGGAACGTGAGGCAGACAAGCTGGAAAATTACAATGGATTGGCTGGTGCAATTGATAAGGTAGATGGAACCAATGTGTCCTGGAAAGGAAATATTCACGCTAACGGGCGCCTTTACATGACCACAAACGGTTTTGCCTGTGGCCAGTATCAACAGTACTTTGGTGGTGTCACTAATCGTTACTCTGTCATGGAGTGGGGAGATGAGAACGGATGGCTAATGTATGTTCACCGTAGAGAGCGGACAACAGCTATAGGAGGTAACATCCAGTTAGTAGTAAACGGACaa                                                                                                                                     >  1:129468/1‑300 (MQ=255)`  
                                                                                                                                                                            `aagcaGACCGTGCGGAACGTGAGGCAGACAAGCTGGAAAATTACAATGGATTGGCTGGTGCAATTGATAAGGTAGATGGAACCGATGTGTACTGGAAAGGAAATATTCACGCTAACGGGCGCCTTTACATGACCACAAACGGTTTTGACTGTGGACAGTATCAACAGTTCTTTGGTGGTGTCACTAATCGTTACTCTGTCATGGAGTGGGGAGATGAGAACTGATGGCTGATGTATGTTCAACGTAGAGAGTGGACAACAGCAATAGGTGGTAACATCCAGTTAGTAGTAAACGGACAAat                                                                                                                                   >  1:103231/1‑301 (MQ=255)`  
                                                                                                                                                                                            `aCGTGAGGCAGACAAGCTGGAAAATTACAATGGATTGGCTGGTGCAATTGATAAGGTAGATGGAACCGATGTGTACTGGAAAGGAAATATTCACGCTAACGGGCGCCTTTACATGACCACAAACGGTTTTGACTGTGGCCAGTATCAACAGTTCTTTGGTGGTGTCACTAATCGTTACTCTGTCATGGAGTGGGGAGATGAGAACGGATGGCTGATGTAGTTTCAACGTAGAGAGTGGACAACAGCGATAGGCGGTAACATCCAGTTAGTAGTAAACGGACAGATCATCACCCAAGGTggg                                                                                                                   >  1:458329/1‑300 (MQ=255)`  
                                                                                                                                                                                              `gTGAGGCAGACAAGCTGGAAAATTACAATGGATTGTCTGGTGCAATTGATAAGGTAGATGGAACCAATGTGTACTGGAAAGGAAATATTCACGCTAACGGGCGCCTTTACATGACCACAAACGGTTTTGACTGTGGCCAGTATAAACAGTTCTTTTGTGGTGTCACTAATCGTTACTCTGTCATGGAGTGGGGAGATGAGAACGGATGGCTGATGTATGTTCAACGTAGAGAGTGGACAACAGCGATAGGCGGTAACATCCAGTTAGTAGTAAACGGACAGatcctgtctcttatacacat                                                                                                                 >  1:238531/1‑284 (MQ=255)`  
                                                                                                                                                                                                  `ggCAGACAAGCTGGAAAATTACAATGGATTGGCTGGTGCAATTGATAAGGTAGATGGAACCAATGTGTACTGGAAAGGAAATATTCACGCTAACGGGCGCCTTTACCTGACCACAACCGGTTTTGACTGTGGCCAGTATCCACAGATCTTTGGTGCTGTCACTAATCTTTACTCTGTCATGGAGTGGGGAGATGAGAACGGATGGCTGATGTATGTTCAACGTAGAGAGTGGACAACAGCGATAGGCGGTAACATCCAGTTAGTAGTAAACGGACAGAACATCACCCAAGGTGGAGCCATg                                                                                                             >  1:467757/1‑301 (MQ=255)`  
                                                                                                                                                                                                              `ggAAAATTACAATGGATTGGCTGGTGCAATTGATAAGGTAGATGGAACCAATGTGTACTGGAAAGGAAATATTCACGCTAACGGGCGCCTTTACATGACCACAAACGGTTTTGACTGTGGCCAGTATCAACAGTTCTTTGGTGGTGTCACTAATCGTTACTCTGTCATGGAGTGGGGAGATGAGAACGGATGGCTGATGTATGTTCAACGTAGAGAGTGGATAACAGCGATAGGCGGTAACATCCAGTTAGTAGTAAACGGACAGATCATCACCCAAGGTGGAGCCATGACCGGTCAGCTa                                                                                                 >  1:135838/1‑301 (MQ=255)`  
                                                                                                                                                                                                                           `ggATTGGCTGGTGCAATTGATAAGGTAGATGGAACCAATGTGTACTGGAAAGGAAATATTCACGTTAACGGGCGCCTTTACATGACCACAAACGGTTTTGACTGTGGCCAGTATCAACAGTTCTTGGGTGGTGTCACTAATCGTTACTCTGTTATGGCGTGGGGCGATGAGAACGGATGGCTGATGTATGTTCATCGTAGAGTGTGGACAACAGCGATAGGCGGTAACATCCAGTTAGTAGTAAACGGACAGATCATCACCCAAGGTGGAGACATGACCGGTCAGCTAAAATTGCAG‑CATg                                                                                   >  1:438977/1‑301 (MQ=255)`  
                                                                                                                                                                                                                               `atataagagacagtGATAAGGTAGATGGAACCGATGTGTACTGGAAAGGAAATATTCACGCTAACGGGCGCCTTTACATGACCACAAACGGTTTTGACTGTGGCCAGTATCAACAGTTCTTTGGTGGTGTCACTAATCGTTACTCTGTCATGGAGTGGGGGGATGAGAACGGATGGCTGATGTATGTTCAACGTAGAGAGTGGACAACAGCGATAGGCGGTAACATCCAGTTAGTAGTAAACGGACAGATCATCACCCAAGGTGGAGCCATGACCGGTCAGCTAAAATTGCAG‑AATGGGCa                                                                               <  1:480818/288‑1 (MQ=255)`  
                                                                                                                                                                                                                                `ggCTGGTGCAATTTATAAGGTAGATGGAACCAATGTGTACTGGAAAGGAAATATTCACGTTAACGGGCGCCTTTACATGCCCACAAACGGTTTTGACTGTGGCCAGTATCAACCGTTCTTTGGTGGTGTCACTAATCGTTACTCTGTCATGGAGTGGGGAGATGAGAACGGCTGGCTGATGTATGTTCAACGTAGAGAGTGGACAACAGCGATAGGCGGTAACATCCCGTTAGTAGCAAACGGACAGATCATCACCCAAGGTGGAGCCATGACCGGTCAACTACAATTGCAG‑AATGGGCAt                                                                              >  1:213489/1‑301 (MQ=255)`  
                                                                                                                                                                                                                                    `ggTGCAATTGATAAGGTAGATGGAACCAATGTGTACTGGAAAGGAAATATTCACGCTAACGGGCGCCTTTACATGACCACAAACGGTTTTGACTGTGGCCAGTATCAACAGTTCTTTGGTGGTGTCACTAATCGTTACTCTGTCATGGAGTGGGGAGATGAGAACGGATGGCTGATGTATGTTCAACGTAGAGAGTGGACAACAGCGATAGGCGGTAACATCCAGTTAGTAGTAAACGGACAGATCATCACCCAAGGTGGAGCCATGACCGGTAAGCTAAAATTGCAG‑AATGGGCATGttc                                                                          <  1:423132/301‑1 (MQ=255)`  
                                                                                                                                                                                                                                    `ggTGCAATTGATAAGGTAGATGGAACCAATGTGTACTGGAAAGGAAATATTCACGCTAACGGGCGCCTTTACATGACCACAAACGGTTTTGACTGTGGCCAGTATCAACAGTTCTTTGGTGGTGTCACTAATCGTTACTCTGTCATGGAGTGGGGAGATGAGAACGGATGGCTGATGTATGTTCAACGTAGAGAGTGGACAACAGCGATAGGCGGTAACAGCCAGTTAGGAGTAAACGGACAGATCATCACCCAAGGTGGAGCCATGACCGGTCAGCAAAAATTGCAG‑AATGTGCATGttt                                                                          >  1:348728/1‑300 (MQ=255)`  
                                                                                                                                                                                                                                     `gTGCAATTGATAAGGTAGATGGAACCAATGTGTACTGGAAAGGAAATATTCACGCTAACGGGCGCCTTTACATGACCACAAACGGTTTTGACTGTGGCCAGTATCAACAGTTCTTTGGTGGTGTCACTAATCGTTACTCTGTCATGGAGTGGGGAGATGAGAACGGATGGCTGATGTATGTTCAACGTAGAGAGTGGACAACAGCGATAGGCGGTAACATCCAGTTAGTAGTAAACGGACAGATCATCACCCAAGGTGGAGCCATGACCGGTCAGCTAAAATTGCAG‑AATGGGCATGttct                                                                         >  1:298446/1‑301 (MQ=255)`  
                                                                                                                                                                                                                                        `cAATTGATAAGGTAGATGGAACCAATGTGTACTGGAAAGGAAATATTCACGCTAACTGGCGCCTTTACATGACCACAAACGGTTTTGACTGTGGCCAGTATCAACAGTTCTTTGGTGGTGTCACTAATCGCTACTCTATCATGGAGTGGGGTGATGAGAACGGATCGCTTATGTATGTTCAACGTAGAGAGTGGACAACAGCGATAGGCGGTAACATCCAGTTAGTAGGAAACGGACAGATCATCACCCAAGGTGTAGACATGACCGGTCAGCTAAAATTGCAG‑AATTGGCATGTTCTTCa                                                                      >  1:234583/1‑301 (MQ=255)`  
                                                                                                                                                                                                                                        `cAATTGATAAGGTAGATGGAACCAATGTGTACTGGAAAGGAAATATTCACGCTAACGGGCGCCTTTACATGACCACAAACGGTTTTGACTGTGGCCAGTATCAACAGTTCTTTGGTGGTGTCACTAATCGTTACTCTGTCATGGAGTGGGGAGATGAGAACGGATGGCTGATGTATGTTCAACGTAGAGAGTGGACAACAGCGATAGGCGGTAACATCCAGTTAGTAGTAAACGGACAGATCATCACCCAAGGTGGAGCCATGACCGGTCAGCTAAAATTGCAG‑AATGGGCATgctgtctc                                                                      >  1:477235/1‑294 (MQ=255)`  
                                                                                                                                                                                                                                          `aTTGATAAGGTAGCTGGAACCAATGTGTACTGGAAAGGAAATATTCACGCTAACGGGCGCCTTTACATGACCACAAACGGTTTTGACTGTGGCCAGTATCAACAGTTCTTTGGTGGTGTCACTAATCGTTACTCTGTCATGGAGTGGGGAGATGAGAACGGATGGCTGATGTTTGTTCAACGTAGAGAGTGGACAACAGCGATAGGCGGTAACATCCAGTTAGTAGTAAAAGGACAGATCATCACACAAGTTGGAGCCATGACCGGTCAGCTAAAATTGCAG‑AATGGGCATGTTCTTCAAt                                                                    >  1:241550/1‑301 (MQ=255)`  
                                                                                                                                                                                                                                           `atacgagaccggATGGAACCAATGTGTACTGTAAAGGAAATATTCACGCTAACGGGCGCCTTTACATGACCACAAACGTTTTTGACTGTGGCCAGTATCAACAGTTCTTTGGTGGTGTCACTAATCGTTACTCTGTCATGGAGTGGGGAGATGAGAACGGATGGCTGATGTATGTTCAACGTAGAGAGTGGACAACAGCGATAGGCGGTAACATCCAGTTAGTAGTAAACGGACAGATCATCACCCAAGGTGGAGCCATGACCGGTCAGCTAAAATTGCAG‑AATGGGCATGTTCTTCAAtt                                                                   <  1:210753/290‑1 (MQ=255)`  
                                                                                                                                                                                                                                             `aagaaagTAGATGGACAAAATGTGTAAAGGAAAGGAAATGTGCACGGTAACGGGAGCCTTTACAGGAGGACAAACGGTTTTGACTGTGGCCAGTATAAACAGTTGTTTGGTGGTGTCAATAATCGTTACTCTGTAATGGAGTGGGGAGATGAAAACGGATGGCTGATTTATGTGCGGCGTAGAGAGTGGACAAAAGCGATAGGCGGTAACATCCAGTTAGTAGTAAACGGACAGATCAGCACCCAAGGTGGAGCCATGACCGGTCAGCTAAAAGTGCAG‑AATGGGCATGTTCTTCAATTag                                                                 <  1:246567/295‑1 (MQ=255)`  
                                                                                                                                                                                                                                                 `aGGTAGATGGAACCAATGTGTACTGGAAAGGAAATATTCACGCTAACGGGCGCCTTTACATGACCACAAACGGTTTTGACTGTGGCCAGTATCAACAGTTCTTTGGTGGTGTCACTAATCGTTACTCTGTCATGGAGTGGGGAGATGAGAACGGATGGCTGATGTATGTTCAACGTAGAGAGTGGACAACAGCGATAGGCGGTAACATCCAGTTAGTAGTAAACGGACAGATCATCACCCAAGGTGGAGCCATGACCGGTCAGCTAAAATTGCAG‑AATGGGCATGTTCTTCAATTAGAGTc                                                             <  1:352401/301‑1 (MQ=255)`  
                                                                                                                                                                                                                                                   `gTAGATGGAACCAATGTGTACTGGAAAGGAAATATTCACGCTAACGGGCGCCTTTACATGACCACAAACGGTTTTGACTGTGGCCAGTATCAACAGTTCTTTGGTGGTGTCACTAATCGTTACTCTGTCATGGAGTGGGGAGATGAGAACGGATGGCTGATGTATGTTCAACGTAGAGAGTGGACAACAGCGATAGGCGGTAACATCCAGTTAGTAGTAAACGGACAGATCATCACCCAAGGTGGAGCCATGACCGGGCAGCTAAAATTGCAG‑AATGGGCATGTTCTTCAATTAGAGTCCg                                                           >  1:181874/1‑301 (MQ=255)`  
                                                                                                                                                                                                                                                      `gtcagAACCAATGTGTACTGGAAAGGAAATATTCACGCTAACGTGCGCCTTTACATGACCACAAACGGTTTTGACTGTGGCCAGTATCAACAGTTCTTTGGTGGTGTCACTAATCGTTACTCTGTCATGGAGTGGGGAGATGAGAACGGATTGCTGATGTATGTTCAACGTAGAGAGTGGACAACAGCGATAGGCGGTAACATCCAGTTAGTAGTAAACGGACAGATCATCACCCAAGGTGGAGCCATGACCGGTCAGCTAAAATTGCAG‑AATGGGCATGTTCTTCAATTAGAGTCCGCAt                                                        >  1:19015/5‑301 (MQ=255)`  
                                                                                                                                                                                                                                                          `gcaCCAATGTGTACTGGAAAGGCCCTCTTCCCGCTCACGGTCGCCTTTCCATGCCCACAAACGGTTTTGACTGTGGCCAGTATCAACAGTTCTTTGGTGGTGTCACTACTCGTTACTCTGTCATGGAGTGGGGAGATGAGAACGGATGGCTGATGTATGTTCAACGTAGAGAGTGGACAACAGCGATAGGCGGTAACATCCAGTTAGTAGTAAACGGACAGATCATCACCCAAGGTGGAGCCATGACCGGTCAGCTAAAATTGCAG‑AATGGGCATGTTCTTCAATTAGAGTCCGCATCCGa                                                    >  1:303372/3‑301 (MQ=255)`  
                                                                                                                                                                                                                                                          `gAACCGATGTGTACTGGAAAGGAAATATTCACGCTAACGGGCGCCTTTACATGACCACAAACGGTTTTGACTGTGGCCAGTATCAACAGTTCTTTTGTGGTGTCACTAATCGTTACTCTGTCATGGAGTGGGGAGATGAGAACGGATGGCTGATGTCTGTTCAACGTAGAGAGTGGACAACAGCGATAGGCGGTAACATCCAGTTAGTAGTAAACGGACAGATCATCACCCAAGGTGGAGCCATGACAGGTCAGCTAAAATTGCAG‑AATGGGCATGTTCTTCAATTATAGTCCGCATCCGa                                                    >  1:107345/1‑301 (MQ=255)`  
                                                                                                                                                                                                                                                                  `gtgtACTGGAAAGGAAATATTCACGCTAACGGGCGCCTTTACCTGACCACAAACGGTTTTGACTGTGGCCAGTATCAACAGTTCTTTGGTGGTGTCACTAATCGTTACTCTGTCATGGAGTGGGGAGATGAGAACGGATGGCTGATGTATGTTCAACGTAGAGAGTGGATAACAGCGATAGGCGGTAACATCCAGTTAGTAGTAAACGGACAGATCATCACCCAAGGTGGAGCCATGACCGGTCAGCTAAAATTGCAG‑AATGGGCATGTTCTTCAATTAGAGTCCGCATCCGACAAGgcgc                                            >  1:357521/1‑301 (MQ=255)`  
                                                                                                                                                                                                                                                                    `gtACTGGAAAGGAAATATTCACGCTAACGGTCGCCTTTACATGACCACAAACGGTTTTGACTGTGGCCAGTATCAACAGTTCTTTGGTGGTGTCACTAATCGTTACTCTGTCATGGAGTGGGGAGATGAGAACGGATGGCTGATGTATGTTCAACGTAGAGAGTGGACAAAAGCGATAGGCGGTAACATCCAGTTAGTAGTAAACGGACAGATCAT‑AACCAAATTGGGGGCATGACCCGTGCTCTTAAAATTCAGTAATGGGCATGTTCTTTATCTAGAGTCCGCATCGAACAGGCCGCat                                          >  1:336610/1‑300 (MQ=255)`  
                                                                                                                                                                                                                                                                    `gtACTGGAAAGGAAATATTCACGCTAACGGGCGCCTTTACATGACCACAAACGGTTTTGACTGTGGCCAGTATCAACAGTTCTTTGGTGGTGTCACTAATCGTTACTCTGTCATGGAGTGGGGAGATGAGAACGGATGGCTGATGTATGTTCAACGTAGAGAGTGGACAACAGCGATAGGCGGTAACATCCAGTTAGTAGTAAACGGACAGATCATCACCCAAGGTGGAGCCATGACCGGTCAGCTAAAATTGCAG‑AATGGGCATGTTCTTCAATTAGAGTCCGCATCCGAcctgtctctt                                          >  1:317647/1‑292 (MQ=255)`  
                                                                                                                                                                                                                                                                       `agggaaaggaaaTATTCACGCTAACGGGCGCCGTTACATGACGACAAACGGTTTTTACTGTGGCCAGTATCAACAGTTCTTTGGTGGTGTCACTAATCGTTACTGTGTCATGGAGTGGGGAGATGAGAACGGAGGGCTGATGTATGTTCAACGTAGAGAGTGGACAACAGCGATAGGCGGTAACATCCAGTTAGTAGTAAACGGACAGATCATCACCCAAGGTGGAGCCATGACCGGTCAGCGAAAATTGCAG‑AATGGGCATGTTCTTCAATTAGAGTCCGCATCCGACAAGGCGCACtat                                       <  1:89848/299‑1 (MQ=255)`  
                                                                                                                                                                                                                                                                           `ggctcggagatgggtataagagacagcatttACATGACCACAAACGGTTTTGACTGTGGCCAGTATCAACAGTTCTTTGGTGGTGTCACTAATCGTTACTCTGTCATGGAGTGGGGAGATGAGAACGGATGGCTGATGTATGTTCAACGTAGAGAGTGTACAACAGCGATAGGCGGTAACATCCAGTTAGTAGTAAACGGACAGATCATCACCCAAGGTGGAGCCATGACCGGTCAGCTAAAATTGCAG‑AATGGGCATGTTCTTCAATTAGAGTCCGCATCCGACAAGGCGCACTATATtc                                   <  1:341892/273‑1 (MQ=255)`  
                                                                                                                                                                                                                                                                           `aaaggaaaTATTCACGCTAACGGGCGCCTTTACCTGAACACAAACGGTTTTGACTGTGGCCAGTATCAACAGTTCTTTGGTGGTGTCACTAATCGTTACTCTGTCATGGAGTGGGGAGATGAGAACGGATGGCTGATGTATGTTCAACGTAGAGAGTGGACAACAGCGATAGGCGGTAACATCCAGTTAGTAGTAAACGGACAGATCATCACCCAAGGTGGAGCCATGACCGGTCAGCTAAAATTGCAG‑AATGGGCATGTTCTTCAATTAGAGTCCGCATCCGACAAGGCGCACTATATtc                                   <  1:405315/301‑1 (MQ=255)`  
                                                                                                                                                                                                                                                                            `aaggaaaTATTCACGCTAACGTGCGCCTTTACATGACCACAAACTGTTTTGACTGTGGCCAGTATCAACAGTTCTTTGGTGTTGTCACTAATCGTTACTCTGTCATGGAGTGGGGAGATGAGAACGGATTGCTGATGTATGTTCAACGTAGAGAGTGGACAACAGCGATAGGCGGTAACATCCAGTTAGTAGTAAACGGACAGATCATCACCCAAGGTGGAGCCATGACCGGTCAGCTAAAATTGCAG‑AATGGGCATGTTCTTCAATTAGAGTCCGCATCCGACAAGGCGCACTATATtct                                  <  1:72960/301‑1 (MQ=255)`  
                                                                                                                                                                                                                                                                              `ggaaaTATTCACGCTAACGGGCGCCTTTACATGACCACAAACGGTTTTGACTGTGGCCAGTATCAACAGTTCTTTGGTGGTGTCACTAATCGTTACTCTGTCATGGAGTGGGGAGATGAGAACGGATGGCTGATGTATGTTCAACGTAGAGAGTGGACAACAGCGATAGGCGGTAACATCCAGTTAGTAGTAAACGGACAGATCATCACCCAAGGTGGAGCCATGACCGGTCAGCTAAAATTGCAG‑CATGGGCATGTTCTTCAATTAGAGTCCGCATCCGACAAGGCGCACTATATtctct                                >  1:453014/1‑299 (MQ=255)`  
                                                                                                                                                                                                                                                                               `gaaaTATTCACGCTAACGGGCGCCTTTACCTGACCACAAACGGTTTTGACTGTGGCCAGTATCAACAGTTATTTGGTGGTGTCACTAATCGTTACTCTGTCATGGAGTGGGGAGATGAGAACGGATGGCTGATGTATGTTCAACGTAGAGAGTGGACAACAGCGATAGGCGGTAACATCCAGTGAGTAGTAAACGGACAGATCATCACACAAGGTGGAGCCATGACCGGTAAGCTAAAATTGCAG‑AATGGGCATGTTCTTCAATTAGAGTCCGCATCCGACAAGGCGCACTATATtctatc                               >  1:346543/1‑301 (MQ=255)`  
                                                                                                                                                                                                                                                                               `gaaaTATTCACGCTAACGGGCGCCTTTACATGACCACAAACGGTTTTGACTGTGGCCAGTATCAACAGTTCTTTGGTGGTGTCACTAATCGTTACTCTGTCATGGAGTGGGGAGATGAGAACGGATGGCTGATGTATGTTCAACGTAGAGAGTGGACAACAGCGATAGGCGGTAACATCCAGTTAGTAGTAAACGGACAGATCATCACCCAAGGTGGAGCCATGAACGGTCAGCTAAAATTGCAG‑AATGGGCATGTTCTTCAATTAGAGTCCGCATCCGACAAGGCGCACTATATtctatc                               >  1:164632/1‑301 (MQ=255)`  
                                                                                                                                                                                                                                                                                `aaaTATTCACGCTAACGGGAGACTTTACATGACCACAACCGGTTTTGACTGTGGCCAGTATCAACTGTTCTTTGGTGGTGTCAATAATCGTTACTCTGTCATGGAGTGGGGAGATGAGAACGGATGGCTGATGTATGTTCAACGTAGAGAGTGGACAACAGCGATAGGCGGTAACATCCAGTTAGTAGTAAACGGACAGATCATCACCCAAGGTGGAGCCATGACCGGTCAGCTAAAATTGCAG‑AATGGGCATGTTCTTCAATTAGAGTCCGCATCCGACAAGGCGCACTATATtctatct                              <  1:195241/301‑1 (MQ=255)`  
                                                                                                                                                                                                                                                                                   `tatTCACGCTAACGGGCGCCTTTACATGACCACAAACGGTTTTGACTGTGGCCAGTATCAACAGTTCTTTGGTGGTGTCACTAATCGTTACTCTGTCATGGAGTGGGGAGATGAGAACGGATGGCTGATGTATGTTCAACGTAGAGAGTGGACAACAGCGATAGGCGGTAACATCCAGTTAGTAGTAAACGGACAGATCATCACCCAAGGTGGAGCCATGACCGGTCAGCTAAAATTTCAG‑AATGTGCATGTTCTTCTCTTAGATTCCGCATCAGACCAGGCGCACTATATTCTAGCCaaa                           >  1:288880/1‑301 (MQ=255)`  
                                                                                                                                                                                                                                                                                                     `ccTTTACATGACCACAAACGGTTTTGACTGTGGCCAGTATCAACAGTTCTTTTGTGGTGTCACTAATCGTTACTCTGTCATGGAGTGGGGAGATGAGAACGGATGGCTGATGTATGTTCAACGTAGAGAGTGGACAACAGCGATAGGCGGTAACATCCAGTTAGTAGTAAACGGACAGATCTTCACCCAAGGTCGAGCCATGACCGGTCCGCTAACATTGCCG‑AATGGTCCTCATCTTCAATTATAGTTCGCATCCGACAAGGCGCAatatatctgtctcctatacacatttccgcgccca         >  1:308954/1‑273 (MQ=255)`  
                                                                                                                                                                                                                                                                                                     `ccTTTACATGACCACAAACGGTTTTGACTGTGGCCAGTATCAACAGTTCTTTGGTGGTGTCACTAATCGTTACTCTGTCATGGAGTGGGGAGATGAGAACGGATGGCTGATGTATGTTCAACGTAGAGAGTGGACAACAGCGATAGGCGGTAACATCCAGTTAGTAGTAAACGGACAGATCATCACCCAAGGTGGAGCCATGACCGGTCAGCTAAAATTGCAG‑AATGGGCATGTTCTTCAATTAGAGTCCGCATCCGACAAGGCGCACTATATTCTATCTAAAGATGGTAACAGGAATAAc         >  1:139650/1‑301 (MQ=255)`  
                                                                                                                                                                                                                                                                                                     `ccTTTACATGACCACAAACGGTTTTGACTGTGGCCAGTATCAACAGTTCTTTGGTGGTGTCACTAATCGTTACTCTGTCATGGAGTGGGGAGATGAGAACGGATGGCTGATGTATGTTCAACGTAGAGAGTGGACAACAGCGATAGGCGGTAACATCCAGTTAGTAGTAAACGGACAGATCATAACCCACGATGGAGCCATGCCCGGTCAGCTAAACTTGAAG‑AATGGGCATGTTCTTCACTTAGAGTCCGCCTCCGCCAAGGCGCACTATCTTCTATTTAAAGATTGTAACACGAATAAc         >  1:94447/1‑301 (MQ=255)`  
                                                                                                                                                                                                                                                                                                      `agtaaCATGACCACAAACGGTTTTTGCTGTGGCAAGTATCAACAGTTCTTTGGTGGTGTCACTAATCGGTACTCTGGCATGGAGTGGGGAGATGAGAACGGATGGCTGATGTAGGTTAAACGTAGAGAGTGAACAACAGCGATAGGCGGTAACAGCCAGTTAGTAGTAAACGGACAGATCATCACCCAAGGTGGAGCCATGAACGGTCAGCTAAAATTGCAG‑AATGGGCATGTTCTTCAATTAGAGTCCGCATCCGACAAGGCGCACTATATTCTATCTAAAGAGGGTAACAGGAATAACt        <  1:381468/297‑1 (MQ=255)`  
                                                                                                                                                                                                                                                                                                            `aTGACTACAAACGGTTTTGACTGTGGCCATTATCAACAGTTCGTTGTTAGTGTCACTAATCGTGACGCTATCATGGAGTGGGGAGAGGAGAACGGATTGCTGATGTATGTTCAACGTATAGAGTTGACAACAGCGATAGGCGGTAACTTCCAGTTAGTAGTAAACGGACAGATCATTACCCAAGGTGGAGCCATGACCGGTCAGCTAAAATTGCAG‑AATGTGCATGTTCTTCAATTATAGTCCTCATCCGACTAGGCGCACTATAATCTATCTAAAGATGGTAACAGGAATAACTGGTACa  <  1:272128/301‑1 (MQ=255)`  

                                                                                                                                                                                                                                                                                                            `|`                                                                                                                                                                                                                                                                                                               
`CAGTGGCGCGATGAGACCAAGGGTTTCCGAGACGAAGCCAAGCGGTTCAAGAATACGGCTGGTCAATACGCTACATCTGCTGGGAACTCTGCTTCCGCTGCGCATCAATCTGAGGTAAACGCTGAGAACTCTGCCACAGCATCCGCTAACTCTGCTCATTTGGCAGAACAGCAAGCAGACCGTGCGGAACGTGAGGCAGACAAGCTGGAAAATTACAATGGATTGGCTGGTGCAATTGATAAGGTAGATGGAACCAATGTGTACTGGAAAGGAAATATTCACGCTAACGGGCGCCTTTACATGACCACAAACGGTTTTGACTGTGGCCAGTATCAACAGTTCTTTGGTGGTGTCACTAATCGTTACTCTGTCATGGAGTGGGGAGATGAGAACGGATGGCTGATGTATGTTCAACGTAGAGAGTGGACAACAGCGATAGGCGGTAACATCCAGTTAGTAGTAAACGGACAGATCATCACCCAAGGTGGAGCCATGACCGGTCAGCTAAAATTGCAG‑AATGGGCATGTTCTTCAATTAGAGTCCGCATCCGACAAGGCGCACTATATTCTATCTAAAGATGGTAACAGGAATAACTGGTACA  >  NC_001604.1/35215‑35815`

  

Alignment Legend

Aligned base mismatch/match (shaded by quality score): `ATCG/ATCG` `< 3 ≤` `ATCG/ATCG` `< 10 ≤` `ATCG/ATCG` `< 18 ≤` `ATCG/ATCG` `< 32 ≤` `ATCG/ATCG` `< 38 ≤` `ATCG/ATCG`

Unaligned base: `atcg`    Masked matching base: `atcg`    Alignment gap: `‑`    Deleted base: `‑`
